# Neuroinflammation in Age-Related Neurodegenerative Diseases: Role of Mitochondrial Oxidative Stress

**DOI:** 10.3390/antiox13121440

**Published:** 2024-11-22

**Authors:** Xenia Abadin, Cristina de Dios, Marlene Zubillaga, Elia Ivars, Margalida Puigròs, Montserrat Marí, Albert Morales, Marisa Vizuete, Javier Vitorica, Ramon Trullas, Anna Colell, Vicente Roca-Agujetas

**Affiliations:** 1 Department of Cell Death and Proliferation, Institut d’Investigacions Biomèdiques de Barcelona (IIBB), Consejo Superior de Investigaciones Científicas (CSIC), Institut d’Investigacions Biomèdiques August Pi i Sunyer (IDIBAPS), 08036 Barcelona, Spain; xenia.abadin@iibb.csic.es (X.A.); marlene.zubillaga@iibb.csic.es (M.Z.); elia.ivars@iibb.csic.es (E.I.); margalida.puigros@iibb.csic.es (M.P.); monmari@clinic.cat (M.M.); albert.morales@iibb.csic.es (A.M.); ramon.trullas@iibb.csic.es (R.T.); 2 Centro de Investigación Biomédica en Red de Enfermedades Neurodegenerativas (CIBERNED), Instituto de Salud Carlos III, 28029 Madrid, Spain; mvizuete@us.es (M.V.); vitorica@us.es (J.V.); 3 Departament de Biomedicina, Facultat de Medicina, Universitat de Barcelona, 08036 Barcelona, Spain; 4 High Technology Unit, Vall d’Hebron Research Institute, 08035 Barcelona, Spain; cristina.dios@vhir.org; 5 Centro de Investigación Biomédica en Red en Enfermedades Hepáticas y Digestivas (CIBEREHD), Instituto de Salud Carlos III, 28029 Madrid, Spain; 6 Department of Biochemistry and Molecular Biology, Faculty of Pharmacy, Universidad de Sevilla, Instituto de Biomedicina de Sevilla (IBiS)-Hospital Universitario Virgen del Rocío/CSIC, 41013 Sevilla, Spain

**Keywords:** inflammasomes, pyroptosis, necroptosis, cuproptosis, ferroptosis, mitochondria, mitochondrial oxidative stress, neuroinflammation

## Abstract

A shared hallmark of age-related neurodegenerative diseases is the chronic activation of innate immune cells, which actively contributes to the neurodegenerative process. In Alzheimer’s disease, this inflammatory milieu exacerbates both amyloid and tau pathology. A similar abnormal inflammatory response has been reported in Parkinson’s disease, with elevated levels of cytokines and other inflammatory intermediates derived from activated glial cells, which promote the progressive loss of nigral dopaminergic neurons. Understanding the causes that support this aberrant inflammatory response has become a topic of growing interest and research in neurodegeneration, with high translational potential. It has been postulated that the phenotypic shift of immune cells towards a proinflammatory state combined with the presence of immunogenic cell death fuels a vicious cycle in which mitochondrial dysfunction plays a central role. Mitochondria and mitochondria-generated reactive oxygen species are downstream effectors of different inflammatory signaling pathways, including inflammasomes. Dysfunctional mitochondria are also recognized as important producers of damage-associated molecular patterns, which can amplify the immune response. Here, we review the major findings highlighting the role of mitochondria as a checkpoint of neuroinflammation and immunogenic cell deaths in neurodegenerative diseases. The knowledge of these processes may help to find new druggable targets to modulate the inflammatory response.

## 1. The Immune System in the Brain and Its Role in Neurodegenerative Disorders

The ability of our organism to discriminate between harmful and innocuous molecules is not a piece of luck. Self-defense is mediated by a complex, well-organized immune system that uses both innate and adaptive mechanisms to recognize and scavenge these harmful elements and secrete cytokines in response to damage. The brain was previously considered an “immune privileged” organ isolated from the peripheral immune system. This statement has been revisited, and the existence of intricate neuroimmune interactions with the involvement of resident and peripheral immunological players that guarantee efficient central nervous system (CNS) surveillance is now recognized [1]. Two brain-resident immune populations have been described: parenchymal microglia and CNS-associated macrophages (CAMs) that are located in specific immunological niches in the CNS borders, including the meninges, the choroid plexus, and the perivascular spaces. These immunological compartments also harbor adaptive immune cells that support healthy brain function, mostly indirectly by releasing cytokines and neurotrophic factors, while limiting exposure to peripheral immune challenges [2]. Alternatively, these cells can access the CNS parenchyma in response to damage signals and provide additional support to resident immune cells [3].

Associated with normal aging, there is an increased infiltration of a variety of peripheral immune cells, including CD4+, CD8+, and CD3+ lymphocytes, natural killer (NK) cells, and myeloid cells, that, together with an abnormal immune response of brain-resident cells, may contribute to maintaining a proinflammatory environment, which is a common feature of all neurodegenerative diseases [4]. The sequence of events that promote the mobilization of peripheral immune cells to the brain remains under debate; however, evidence points to age-dependent changes in microglia with the release of chemotactic signals and the blood–brain barrier (BBB)’s integrity loss as crucial factors [5,6]. Aging has been described as the primary risk factor for most neurodegenerative diseases, including Alzheimer’s disease (AD), Parkinson’s disease (PD), Huntington’s disease (HD), and frontotemporal lobar dementia (FTD) [7]. Hallmarks of cellular aging, such as cellular senescence, genomic instability, dysfunctional mitochondria, and defective proteostasis mechanisms (macroautophagy, hereafter referred to as autophagy), are shared by all neurodegenerative diseases [8]. Alteration of these cellular pathways results in the accumulation of specific pathogenic proteins, for instance, extracellular amyloid beta (Aβ) plaques and intraneuronal tau-containing neurofibrillary tangles (NFTs) in AD or misfolded α-synuclein (α-syn) in PD, which are ultimately responsible for the clinical and pathological diversity of disease phenotypes [9]. Neuroinflammation was initially considered a bystander in neurodegeneration; however, there are now growing data highlighting the key role of a dysfunctional immune system in the neurodegenerative process. This is further supported by the discovery of a high number of immune-related genetic mutations identified as risk factors for neurodegeneration [10,11]. In this review, we will outline new findings addressing the aberrant mechanisms and affected inflammatory signaling pathways leading to neuroinflammation. We will mainly focus on reviewing studies that link neuroinflammation to AD and PD, the most prevalent age-related neurodegenerative disorders, and discuss the involvement of mitochondria and mitochondrial oxidative stress.

### The Contribution of the Innate–Adaptive Immunity to Neurodegeneration

Aging is known to affect the cross-talk between adaptive and innate immunity in the brain. Disruption of this relationship has consistently been associated with age-related neurodegenerative diseases. In mouse models of AD, an imbalance between type I and type II interferons (IFNs) has been reported in the choroid plexus, resulting in defective immune surveillance [12]. The role of adaptive immunity in neurodegeneration remains poorly understood, exhibiting both beneficial and detrimental functions. Marsh et al. have shown that immune-deficient 5xfAD (Rag-5xfAD) mice lacking IgG-producing B cells display impaired microglial phagocytosis accompanied by increased Aβ deposition [13]. In these mice, defective plaque clearance is restored by the administration of non-specific IgGs, which are recognized by Fcγ receptors on microglia and activate the phagocytic Src/spleen tyrosine kinase (Syk)/phosphatidylinositol 3-kinase signal transduction pathway [13]. However, IgG influx into the brain is a double-edged sword, potentially leading to harmful effects. In the presence of Aβ or α-syn, IgGs can trigger an immune complex-mediated inflammatory response associated with neuronal death by activating microglia through Fcγ receptors [14,15]. Additionally, the genetic loss of B cells, and thus IgGs, in various AD mouse models has been shown to reduce Aβ deposition and the presence of reactive microglia, improving disease symptoms [16]. Recent data from postmortem studies also confirm the presence of monocyte-derived cells in prefrontal cortices affected by PD [17] and in the hippocampal parenchyma during advanced stages of AD [18]. Whether this infiltration is beneficial or detrimental to the neurodegenerative process remains to be fully elucidated. In APPswe/PSEN1dE1 mice, monocyte extravasation into the brain, facilitated by peripheral immune modulation, mitigates AD pathology [19]. In contrast, in a mouse model of PD, monocyte invasion has been described as required for α-syn-induced inflammation and neuronal death [20]. Growing evidence indicates that excessive extravasation of lymphocytes and neutrophils from the bloodstream results in cytotoxicity and perpetuates a proinflammatory state in neurodegenerative disorders [21,22,23]. The close connection between innate and adaptive immunity in neurodegeneration is highlighted in a study by Chen et al. The study shows that in mice with tauopathy but not amyloid, there is a distinct immune response. In this response, T cells actively engage with disease-associated microglia subgroups to promote tau-mediated brain atrophy [24]. Furthermore, in these mice, microglia depletion abolishes T cell infiltration, while depletion of T cells blocks microglia activation and ameliorates neurodegeneration and cognition. Similar dysregulated peripheral immunity has been described in patients with AD, PD, and Lewy body dementia, showing clonally expanded peripheral T cells that migrate to and accumulate in the CNS parenchyma and cerebrospinal fluid (CSF) and correlate with disease progression [25,26,27,28].

#### The Role of Microglia in the Neurodegenerative Process

Microglia constitute a heterogeneous population with essential roles in brain development and maintenance [29]. Under homeostatic conditions, microglial phagocytosis and the secretion of precise neurotrophic factors and cytokines have been shown to regulate synaptic plasticity and neuronal activity [30,31,32]. These resident macrophages are also considered the sentinels of the brain, constantly scanning the environment to quickly respond to different insults [33]. Upon immune challenge, microglia tend to adopt an amoeboid morphology accompanied by increased phagocytic activity and production of cytokines and reactive oxygen species (ROS) aimed at resolving these threats to homeostasis. Microglia were traditionally classified according to peripheral macrophage terminology as M1 (proinflammatory and neurotoxic type) and M2 (protective type), a dualistic classification now recognized as extremely simplistic. Recent advances in single-cell genomic technologies support the existence of multiple states of microglial activation with different gene expression signatures that are tailored to the stages and severity of a pathological process [34]. Indeed, specific genetic profiles have been associated with neurodegeneration. Initial works in AD mouse models identified triggering receptors expressed on myeloid cells 2 (TREM2)-dependent transcriptional profiles, termed neurodegenerative microglial (MGnD) or disease-associated microglia (DAM) signatures, in sub-clusters of microglia located near Aβ plaques and exposed to neuron or myelin debris [35,36]. These DAM-like signatures have later been shown to coexist with other more neurotoxic profiles associated with tau pathology [37]. Furthermore, their presence has been extended to other neurodegenerative diseases, including amyotrophic lateral sclerosis (ALS) and multiple sclerosis (MS) [38]. Similar microglial heterogeneity has been described in PD [39], with a midbrain-enriched microglia subset that displays transcriptional features of inflammatory and reactive microglia. The key role of microglia in the etiology of age-related neurodegenerative diseases is further substantiated by recent genome-wide association studies (GWASs) that identify genes mainly expressed in microglia in the genetic risk score for AD and related dementias [10,11]. Despite these findings, how the complexity of brain immune network responses is regulated and what promotes the gain of neurotoxic functions of microglia during pathology is largely unknown.

Activation of microglial phagocytosis has been shown to slow AD pathology and cognitive impairment [40,41]. The protective function of microglia benefits from astrocyte-sourced interleukin-3 (IL-3) that stimulates its capacity to cluster and eliminate Aβ and tau aggregates [42]. In contrast, inefficient clearance, aggravated by defective autophagy, has been shown to facilitate the spreading of Aβ and tau seeds [43,44] and promote senile plaque formation [45,46]. Likewise, in mice that express human α-syn, disruption of autophagy in microglia has been shown to favor the accumulation of misfolded α-syn and cause midbrain dopaminergic neuron degeneration [47]. Dysfunctional microglia may also participate in the intercellular propagation and dissemination of protopathic seeds through extracellular vesicle (EV) secretion [48,49,50]. Concordantly, in PS19 mice that express mutant human tau, suppression of tau-containing EVs by pharmacological therapy significantly reduces tau pathology and improves cognitive impairment [51].

Altered microglia phagocytosis in neurodegeneration is part of an aberrant immune response, accompanied by the release of proinflammatory cytokines [52,53,54]. The inflammatory response is initiated when pattern-recognition receptors (PRRs) sense danger signals, which can be categorized into pathogen-associated molecular patterns (PAMPs), immune-responsive molecules from microorganisms, and damage-associated molecular patterns (DAMPs), intracellular molecules released in response to injury [55]. There are membrane-bond PRRs, such as toll-like receptors (TLRs), and cytosol-localized PRRs, such as nucleotide-binding domain and leucine-rich repeat-containing receptors (NLRs), that assemble into inflammasome complexes [56]. Persistent inflammasome activation is thought to play a crucial role in creating a chronic inflammatory environment and eventual neurodegeneration. In addition to Aβ and other misfolded or aggregated proteins related to neurodegenerative diseases, mitochondrial oxidative stress has been shown to trigger inflammasome activation. Recently, Peruzzotti-Jametti et al., using a preclinical model of MS disease and a multi-omic approach, have identified a molecular signature that sustains microglia activation by inducing complex I-mediated ROS generation coupled with increased expression of inflammasome-related proteins [57]. The mechanisms involved in inflammasome assembly and regulation, particularly the role of mitochondria and mitochondrial oxidative stress, are becoming better understood. These new insights will be summarized in the following sections.

## 2. Mitochondrial Control of the Inflammatory Response

Mitochondria are very dynamic organelles, crucial for cell function and survival [58]. In addition to oxidative phosphorylation (OXPHOS) metabolism and energy generation, mitochondria are involved in multiple cellular processes, including redox signaling, stress response, thermogenesis, and calcium homeostasis. During mitochondrial respiration, the electron transport chain (ETC) makes this organelle the central producer of cellular ROS. Mitochondrial ROS (mtROS), along with other mitochondrial constituents, such as mitochondrial DNA (mtDNA), cardiolipin, and metabolic byproducts released into the cytosol or extracellular environment under cellular stress, have been identified as key regulators of innate immunity [59]. Accordingly, the term “mito-inflammation” has recently been coined to refer to mitochondrial involvement in the inflammatory signaling cascade. Mitochondria are sources of mitochondrial DAMPs but can also act downstream in intracellular signaling pathways triggered by external PAMPs or DAMPs [60]. ROS produced by mitochondria activate redox-sensitive transcription factors, such as nuclear factor kappa B (NF-κB) and activator protein-1 (AP-1), that increase the expression of genes related to inflammation [61]. Mitochondria are also the target of self-generated ROS, especially newly synthesized mtDNA before being packaged into nucleoid structures due to its proximity to the ETC [62]. Oxidized DNA fragments are more prone to being released and triggering a stronger immune response [63]. Cytosolic mtDNA can trigger inflammasome assembly [64,65] and the induction of the cyclic GMP–AMP synthase (cGAS)–stimulator of interferon genes (STING) pathway [64,66].

### The mtDNA as a Danger Signal That Activates the cGAS–STING Signaling Pathway

**Figure 1:**
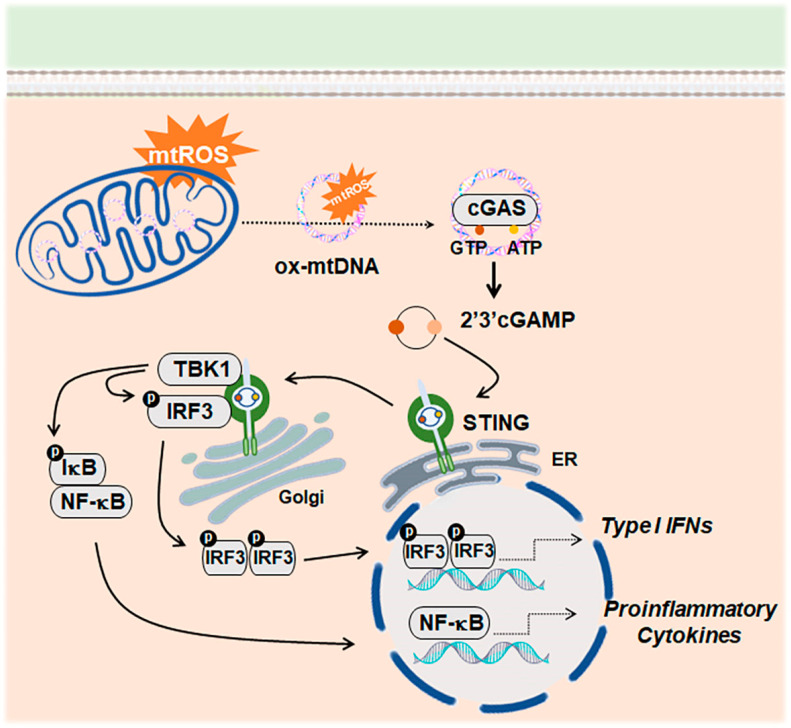
Activation of the cGAS–STING signaling pathway by cytosolic mtDNA. Mitochondrial oxidative stress favors the release of mtDNA. In the cytosol, mtDNA is recognized by cGAS, which then assembles into dimers and catalyzes the synthesis of cGAMP from ATP and GTP. cGAMP initiates the signaling cascade by binding to the ER-associated adaptor protein STING, which translocates to the Golgi apparatus, where it recruits and activates TBK1. TBK1 phosphorylates IRF3, which, in turn, targets the nucleus and transcribes interferon-stimulated genes. TBK1 can also relieve the inhibition of NF-κB, triggering the transcription of gene-encoding proinflammatory cytokines [72]. Abbreviations: ER, endoplasmic reticulum; cGAMP, cyclic 2′,3′-cyclic guanosine monophosphate; cGAS, cyclic GMP–AMP synthase; IRF3, interferon regulatory factor 3; NF-κB, nuclear factor kappa B; STING, stimulator of interferon genes; TBK1, TANK-binding kinase.

The mtDNA can exit mitochondria under cellular stress through different mechanisms, such as the formation of BCL2 antagonist/killer 1 (BAK1) and BCL2-associated X, apoptosis regulator (BAX) macropores [67], or the coordinated participation of mitochondrial permeability transition pore (PTP) with voltage-dependent anion channel 1 (VDAC1) [64,68]. The mtDNA leakage is also promoted by abnormal packaging of the mtDNA due to transcription factor A, mitochondrial (TFAM), or ATPase family AAA domain containing 3A (ATAD3A) deficiency [69,70,71]. Moreover, under mtDNA replication stress conditions, DNA-containing nucleoids have been shown to exit mitochondria into endosomes that ultimately become leaky [69]. Cytosolic mtDNA is sensed by cGAS, linking the recognition of misplaced self-DNA to the start of a type I IFN immune response [72] (Figure 1).

The induction of the c-GAS–STING pathway is controlled by the ER-resident three prime repair exonuclease 1 (TREX1), which breaks down unwanted cytosolic DNA [73]. Remarkably, oxidized DNA has been found to resist degradation by TREX1, leading to its accumulation in the cytosol and the activation of cGAS [74]. Similarly, a mtDNA-mediated induction of the cGAS-dependent immune response has been observed when mitophagy is disrupted [75].

The cGAS–STING signaling pathway has been postulated as a critical driver of aging-related inflammation, which could be therapeutically targeted to halt the neurodegenerative processes in the aged brain [76,77]. Activation of cGAS–STING signaling has been reported in AD mouse brains and human AD fibroblasts [78]. Furthermore, administration of a STING inhibitor ameliorates pathogenesis in 5xfAD mice [79]. Increased cGAS–STING activity has also been associated with neurodegeneration arising from α-synucleinopathies, including PD [80]. cGAS-initiated signaling contributes to the microglial inflammatory response in the mouse model of PD induced by the mitochondrial toxin 1-methyl- 4-phenyl-1, 2, 3, 6-tetrahydropyridine (MPTP) [81]. Notably, a study by Sliter et al. shows that the combination of mtDNA mutational stress with the absence of mitophagy-related proteins leads to dopaminergic neurodegeneration and motor defects that are rescued by genetic inactivation of STING, thus linking mito-inflammation and the cGAS–STING pathway with the onset of PD-like pathology [75].

## 3. Inflammasome Signaling in Neuroinflammation

**Figure 2:**
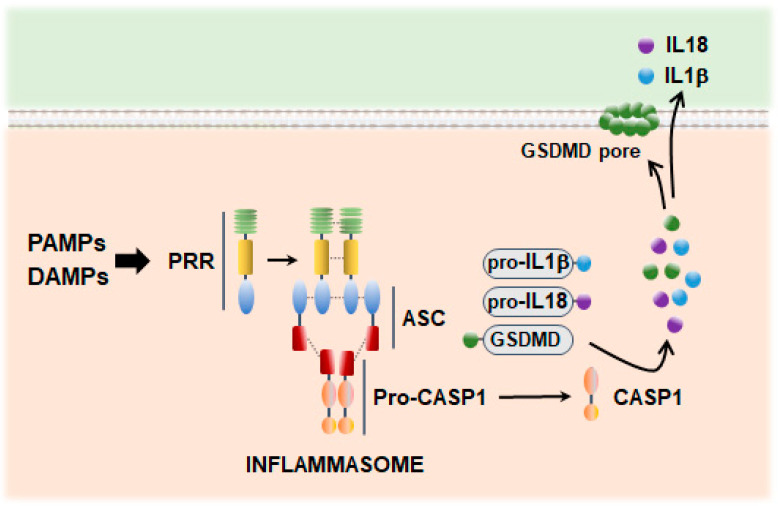
Inflammasome induction. Inflammasome assembly can be triggered by a repertoire of PPRs that detect and bind microbial ligands (PAMPs) and endogenous molecules exposed under stress conditions (DAMPs). The activated sensor then oligomerizes with the adapter protein ASC through PYD interactions and recruits the CASP1 zymogen, which undergoes auto-proteolysis to generate catalytically active CASP1. Mature CASP1 then cleaves and activates the inflammatory cytokines pro-IL-1β and pro-IL-18 and the pore-forming protein GSDMD. Abbreviations: ASC, associated speck-like protein containing a CARD; CASP1, caspase 1; DAMPs, damage-associated molecular patterns; GSDMD, gasdermin D; PAMPs, pathogen-associated molecular patterns; PPRs, pattern recognition receptors; PYD, pyrin domain.

First described by Martinon et al. [82], inflammasomes are multiprotein complexes that act as cytosolic scaffolds to recruit and oligomerize the cysteine protease caspase 1 (CASP1). The function of inflammasomes is best characterized in myeloid lineages where CASP1 catalyzes the maturation of inflammatory cytokines, which are released into the extracellular space and promote both local and systemic immune responses (Figure 2). Alternatively, inflammasome activation can cleave the pore-forming protein gasdermin D (GSDMD), the executor of a lytic mode of cell death termed pyroptosis (Figure 2). Inflammasome proteins have been observed in the CNS, usually in monocytes and microglia responding to acute infection or cell loss, as occurs during neurodegeneration. Although less understood, activated inflammasome signaling has also been reported in neurons linked to pyroptosis [83], providing a mechanism through which neurons may initiate or influence inflammation.

Inflammasomes are constituted by three main components: a sensor consisting of a PRR with a pyrin domain (PYD) and/or a caspase recruitment domain (CARD), the adaptor apoptosis-associated speck-like protein containing a CARD (PYCARD, also known as ASC), and the zymogen of CASP1 (pro-CASP1) (Figure 2). CASP1 consists of an N-terminal CARD, a CARD domain linker, the p20 subunit containing the catalytic sides, an inter-domain linker (IDL), and the p10 subunit involved in dimerization. Mechanistically speaking, it has been proposed that PRR clustering promotes a nucleation-induced polymerization of pro-CASP1 into filaments, facilitating the protease zymogens to undergo proximity-induced autoactivation [84]. The autocatalytic cleavage of pro-CASP1 results in two subunits that form an active p10/p20 tetramer [85], which has been proposed as responsible for the processing of pro-IL-1β and pro-IL-18 [86] and GSDMD (Figure 2). Additionally, CASP1 may also induce NF-κB-mediated pathways [87,88]. Recently, using a biotin-labeled probe that covalently binds to active CASP1, Boucher et al. have questioned the catalytic role of the p20/p10 tetramer under physiological conditions [89]. The study demonstrates that CASP1 clustering on inflammasomes produces active p46 dimers that self-process at the IDL to generate a second active species (p33/p10), which remains associated with the inflammasome. The activity of p33/p10 is blocked by a second self-cleavage event, which removes the CARD domain, releasing a highly unstable dimer (p20/p10) that terminates CASP1 signaling. Notably, the full-length dimeric p46 can effectively process GSDMD but not IL-1β, while p33/p10 can cleave both GSDMD and IL-1β.

Pyroptotic death and cytokine secretion are often simultaneous outcomes of inflammasome activation. However, these events may be uncoupled under certain circumstances [90]. GSDMD sublytic pore formation or GSDMD-independent pathways involving unconventional secretion could result in predominant cytokine uncoupling, whereas regulation of CASP1 cleavage dynamics, which can generate active species with different activities in terms of GSDMD or IL-1β processing, could lead to pyroptosis uncoupling. Recently, in macrophages, the expression of the Toll-1L-1R protein SARM has been identified as a key regulatory player [91]. Nonetheless, the underlying mechanisms of uncoupling remain mostly elusive.

### 3.1. Inflammasome Scaffold Proteins

**Figure 3:**
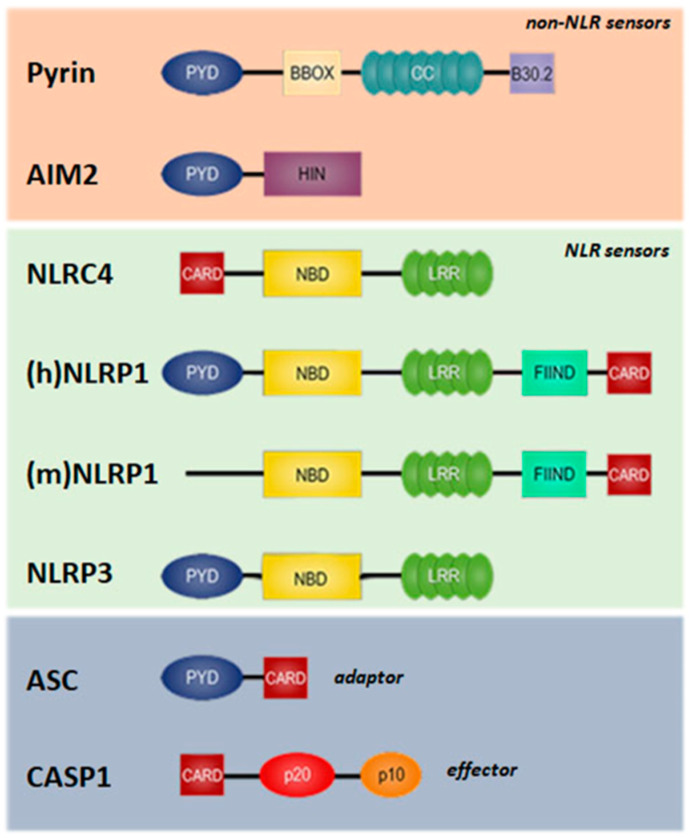
Inflammasome components and their domain architecture. Members of the NLR receptor family contain NBD and LRR motifs. NLR family members may be further subdivided into NLRP (with a PYD motif) and NLRC (with a CARD motif). The AIM2 receptor is composed of a PYD and a dsDNA-binding Hin-200/HIN domain. Finally, the inflammasome scaffold protein pyrin consists of PYD, BBOX, and CC domains that are followed by a carboxy-terminal B30.2 domain in the human but not its murine ortholog. NLRP3, human (h)NLRP1, AIM2, and pyrin, containing a PYD, bind to the PYD of ASC, allowing the ASC to activate CASP1 by interacting with the CARD of pro-CASP1. Card-containing sensors such as mouse (m)NLRP1 and NLRC4 activate CASP1 by directly binding the CARD of pro-CASP1 without ASC or binding the paired ASC scaffold. Abbreviations: AIM2, absent in melanoma 2; ASC, associated speck-like protein containing a CARD; BBOX, B-Box-type zinc finger; CASP1, caspase 1; CARD, caspase recruitment domain; CC, coiled coil; HIN/Hin-200, hematopoietic interferon-inducible nuclear protein with a 200 amino acid repeat; NBD, nucleotide-binding domain; NLR, nucleotide-binding domain and leucine-rich repeat; LRR, leucine-rich repeat; PYD, pyrin domain.

The NLR family members were the first sensor proteins identified in inflammasome formation (Figure 3). Recently, other families of inflammasome-associated PRRs have been described. These last include absent in melanoma 2 (AIM2)-like receptors (ALRs) that respond to self and pathogen double-stranded (ds)DNA molecules and pyrin receptors (also known as tripartite motif-containing (TRIM) 20), which assemble in response to perturbations of cytoplasmic homeostasis and cytoskeletal dynamics caused by infection (Figure 3). Nonetheless, the majority of inflammasomes rely on NLR proteins for pathogen sensing and scaffold assembly. All NLR sensors contain a central nucleotide-binding domain (NBD/NACHT) responsible for their self-oligomerization, a leucine-rich repeats (LRRs) domain crucial in the recognition of ligands (PAMPs and DAMPs), and signaling domains (PYR and CARD) that enable the recruitment of pro-CASP1 [92] (Figure 3). Major inflammasome-forming members are NLRP1, NLRP3, and NLRC4. Other atypical members of the family are NLRP2, NLRP6, NLRP7, NLRP12, and NLRC5, which, although less characterized, can also form an inflammasome complex [93]. Pro-CASP1 can directly interact with NLRC4 and mouse (m)NLRP1, through the CARD motifs, while in PYD-containing members, such as NLRP3 and human (h)NLRP1, the cysteine protease is recruited indirectly through homotypic interactions with the bipartite PYD/CARD adaptor ASC [94].

The NLRP3 is the most extensively studied inflammasome. Canonical activation of the NLRP3 inflammasome requires a priming step with the transcriptional upregulation of NLRP3, CASP1, and IL-1β/IL-18 pro-forms [95,96]. The activation of the NLRP3 inflammasome is thought to be tightly associated with the regulator never in mitosis A (NIMA)-related kinase 7 (NEK7) [97]. Additionally, recent research indicates that post-translational modifications of NLRP3, such as acetylation and phosphorylation, are necessary for full activation of the complex [95,98,99]. These priming mechanisms may serve as a checkpoint to prevent accidental activation of NLRP3, which is the sensor that responds to a wide spectrum of infection and stress-associated signals [100]. The exact molecular mechanism that triggers NLRP3 activation in response to such diverse signals is still under investigation. Muñoz-Planillo et al. proposed K^+^ efflux through purinergic receptor P2X 7 (P2RX7) channels as a universal requirement for NLRP3 activation [101], which is accompanied by the loss of lysosomal integrity [102]. This view has been challenged later by studies showing that NLRP3 activation can be triggered independently of K^+^ efflux by small-molecule TLR7 agonists that target mitochondrial complex I and produce mtROS [103].

### 3.2. Dysfunctional Mitochondria and Mitochondrial ROS as Drivers of Inflammasome Activation

**Figure 4:**
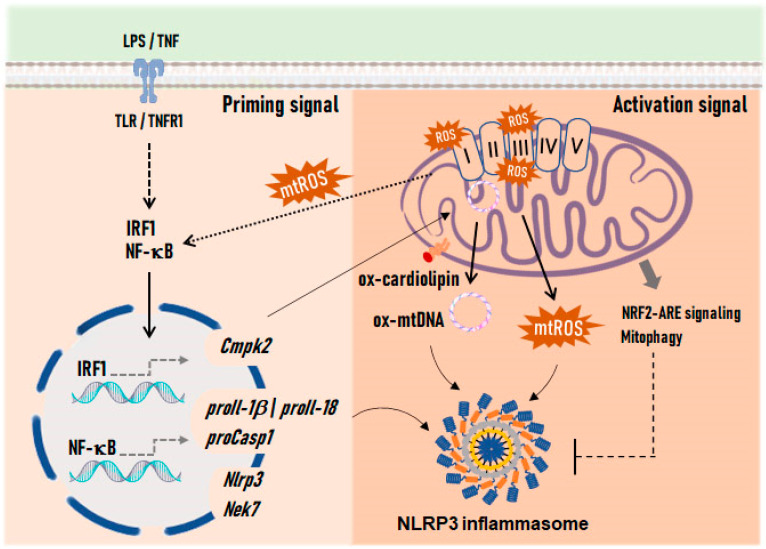
NLRP3 inflammasome induction by mtROS. The mtROS generated in complexes I and III of the electron transport chain confer the priming signal necessary for the NLRP3 inflammasome activation. The cytosolic spread of mtROS activates redox-sensitive transcription factors, such as NF-κB, which stimulate the expression of inflammasome-related genes. Additionally, excessive production of mtROS causes oxidative modifications of mitochondrial proteins, mtDNA, and membrane lipids such as cardiolipin, which favor inflammasome assembly. ox-mtDNA release requires a TLR-dependent priming signal that triggers mtDNA replication through IRF1-mediated induction of CMPK2, an enzyme responsible for dNTPs supply. Dysfunctional mitochondria also engage the NRF2-ARE signaling pathway and mitophagy, which inhibit inflammasome assembly. Abbreviations: ARE, antioxidant response element; CMPK2, cytidine/uridine monophosphate kinase 2; IRF1, interferon regulatory factor 1; NF-κB, nuclear factor kappa B; NRF2, nuclear factor-erythroid 2 (NFE2) p45-related factor 2; dNTPs, deoxynucleotide triphosphates; TLR, toll-like receptor.

NLRP3 inflammasome signaling has been consistently related to mitochondrial dysfunction and oxidative stress (Figure 4). Accordingly, mitophagy induction prevents inflammasome assembly [104], while mtROS serves as a priming signal [61]. On the other hand, the increase in mtROS promotes the binding of NEK7 with NLRP3 [97,103] and facilitates the formation of the complex in mitochondria-associated membranes (MAMs) of dysfunctional mitochondria [105]. This process is suppressed when mitophagy is stimulated or when VDAC2, an isoform of the mitochondrial carrier linked to mtROS regulation, is silenced [105,106,107]. Additionally, it has been described that Ca^2+^ mobilization from the endoplasmic reticulum (ER) enhances inflammasome activation by inflicting damage to mitochondria [108]. Furthermore, optimal NLRP3 inflammasome signaling has been shown to require the interaction between NLRP3 and thioredoxin-interacting protein (TXNIP), a nuclear protein that relocates to mitochondria during oxidative stress [109,110]. In cerebral ischemia-reperfusion injury, the NLRP3-TXNIP binding and subsequent inflammasome activation are prevented by NFE2-like bZIP transcription factor 2 (NFE2L2, also known as NRF2), a master regulator of the cellular antioxidant system, which upregulates the expression levels of thioredoxin 1 (TRX1), a binding partner of TXNIP [111]. On the other hand, ROS-mediated externalization of cardiolipin to the outer membrane has been reported to promote the binding between NLRP3 and CASP1, which assemble upon ASC recruitment to the mitochondrial surface [112,113]. Externalized cardiolipin can also bind inflammasome-activated GSDMD, a necessary event for pyroptosis to proceed [114]. GSDMD permeabilizes the mitochondrial membranes, facilitating mtROS generation and the release of mitochondrial proteins and DNA. In turn, as indicated previously, the NLRP3 inflammasome can be activated by ox-mtDNA released into the cytosol [64,65] and is downregulated by autophagy and mitochondrial integrity preservation [115]. Zhong et al. described that NLRP3 priming relies on the induction of new mtDNA synthesis, downstream of TLR4 engagement, which ultimately triggers an interferon regulatory factor 1 (IRF1)-dependent expression of the mitochondrial nucleoside cytidine/uridine monophosphate kinase 2 (CMPK2) [116] (Figure 4). The resulting increase in CMPK2 levels regulates mtDNA replication by supplying the required deoxyribonucleotides. Cytosolic mtDNA also enables AIM2 inflammasome assembly [117]. Notably, while the NLRP3 inflammasome shows a preferential response to oxidized DNA [65], AIM2-containing counterparts have been suggested to primarily recognize non-oxidized DNA [116].

### 3.3. GSDM-Mediated Inflammatory Cell Death: Pyroptosis

As part of the innate immune response, inflammasomes can also trigger a caspase-dependent cell death that prevents pathogen replication and activates phagocytic immune cells. First coined by Cookson and Brennan [118], this type of programmed necrosis called pyroptosis is featured by cell swelling and the appearance of protrusions on the plasma membrane before its rupture. Pyroptosis initiates after CASP1-mediated cleavage of GSDMD. The proteolytic processing of GSDMD generates a 31 kDa N-terminal domain, which aggregates to form 10–15 nm diameter pores in the cell membrane [119]. The pyroptotic pore enables the release of the proinflammatory cytokines matured by the inflammasome [120] and causes the loss of osmotic gradient, which ultimately leads to cell death, with or without cell lysis [121]. Remarkably, recent research has revealed that the pore-forming activity of GSDM is redox-sensitive [122], highlighting the crucial role of ROS in regulating inflammasome-mediated inflammatory response at multiple levels. The mtROS-mediated oxidative modification of specific cysteine residues promotes GSDMD cleavage by CASP1 [123]. Additionally, mitochondrial oxidative stress, induced by the mechanistic target of rapamycin kinase (MTOR) signaling pathway, has been shown to stimulate pore formation downstream of GSDM cleavage [124]. Likewise, it has been described that mtROS controls the palmitoylation of C191, which is crucial for GSDMD oligomerization [125]. On the other hand, cleaved GSDM can also permeabilize the mitochondrial membrane, resulting in increased mtROS and release of mtDNA, which, in turn, can trigger GSDM oligomerization [126,127].

Pyroptosis has been implicated in the inflammatory pathogenesis of multiple neurological diseases. Preclinical and clinical data provide evidence of GSDMD-mediated pyroptosis in age-related neurodegenerative diseases [54,83,128]. On the other hand, recent research using experimental autoimmune encephalomyelitis (EAE) models indicates that pyroptosis in peripheral myeloid cells may contribute to MS pathogenesis by providing essential signals to engage a T cell immune response [129]. Furthermore, GSDMD inhibitors have been shown to alleviate inflammation and disrupt EAE development [130]. The occurrence of GSDMD-mediated pyroptosis has also been documented in glial and brain microvascular endothelial cells across different ischemic brain injury models [131,132]. There is also evidence that pyroptosis-induced inflammation plays a role in secondary damage in CNS injuries, such as traumatic brain injury and spinal cord injury, resulting in prolonged neuroinflammation and functional decline. Studies indicate that inhibiting pyroptosis, particularly by targeting the NLRP3/CASP1 pathway, could provide neuroprotective effects in these conditions [133].

### 3.4. Inflammasome-Mediated Neuroinflammation in Alzheimer’s and Parkinson’s Disease

Genetic and pharmacologic evidence demonstrates the involvement of the NLR-dependent regulation of inflammation in the progression of different neurodegenerative diseases [134]. In 2008, Halle et al. described the NLRP3 inflammasome activation by fibrillar Aβ in primary microglia cultures [135], which was replicated later using soluble Aβ oligomers [136] and tau seeds [137]. Notably, in cultured cortical neurons, Aβ exposure causes an NLRP1-mediated pyroptosis [83]. Activated microglia can, in turn, release ASC specks that interact with extracellular Aβ, showing cross-seeding and inflammatory boosting activities [45,138]. Similarly, α-syn aggregates in microglia serve as a priming signal via TLR2 ligation and trigger NLRP3 inflammasome assembly associated with ROS production and impaired lysosomal function [139,140]. Conversely, blockage of the NLRP3-mediated signaling pathway restores microglial phagocytosis of the toxic aggregates [137,139].

Markers of inflammasome activation, such as cleaved CASP1 and GSDMD, have been found in brain and CSF samples from AD individuals and transgenic mice [54,128]. Likewise, increased expression of inflammasome-related proteins has been reported in the substantia nigra of patients with PD [52,141]. Inflammasome activation is also observed in the blood cells and correlates with disease progression [142], which has led to their consideration as potential early biomarkers of PD [143].

The involvement of the NLRP3 inflammasome in promoting chronic brain inflammation and neurodegeneration is further supported by preclinical studies, which show that NLRP3 ablation in APPswe/PSEN1dE1 mice induces a microglial shift toward an anti-inflammatory phenotype, associated with lower Aβ deposition and improvement in cognitive decline [54]. A similar reduction in Aβ pathology has been reported in *Nlrp1* knock-out mice [83,144]. Along the same lines, pharmacological inhibition of NLRP3 [145] and GSDMD [146] attenuates pathological symptoms in PD mice. Consistently, mice lacking *Nlrp3* or *Casp1* are resistant to developing PD-like neurodegeneration evoked by neurotoxins that target mitochondria [147,148,149,150]. Moreover, the NLRP3-mediated inflammatory response is blunted when classical PD mitochondrial neurotoxicants are combined with mitochondria-targeted antioxidants [151]. Strong evidence for the role of mitochondria in regulating neuroinflammation is further provided by our recent study showing a differential inflammatory response in microglia and neuronal cells that depends on their mitochondrial antioxidant capacity [152]. In early works, using APPswe/PSEN1dE1 mice overexpressing the sterol regulatory element-binding transcription factor 2 (SREBF2), we demonstrated that elevated brain cholesterol levels accelerate and worsen the pathological features of Alzheimer’s disease, including neuroinflammation [153]. We next have shown that intracellular cholesterol enrichment in microglia triggers a neuroprotective profile mediated by the NLRP3 inflammasome assembly [152]. After the bacterial toxins challenge, cholesterol-enriched microglia display an enhanced release of neurotrophic factors such as nerve growth factor (NGF) and brain-derived neurotrophic factor (BDNF), higher phagocytic capacity, and increased release of encapsulated Il-1β. Moreover, conditioned media from these cells significantly blunt the loss of viability of neuronal cells exposed to Aβ. In contrast, in neuronal cells, increased cholesterol levels compromise the mitochondrial antioxidant defense [152]. In these cells, the exacerbated oxidative stress after Aβ exposure leads to pyroptosis. Pyroptotic neurons, in turn, can inhibit microglia phagocytosis, potentially establishing a proinflammatory vicious cycle.

## 4. Role of Immunogenic Cell Death as a Contributor to Neurodegeneration

There is a close relationship between inflammation and cell death. When necrotic cells lose their cell membrane integrity, they release DAMPs that can be recognized by different cellular receptors, leading to a sterile inflammatory response. In recent decades, different types of regulated necrosis-like death have been described [154]. Understanding the mechanisms that regulate this proinflammatory cell death in neurodegeneration has become a field of intense study. As mentioned earlier, inflammasome induction can trigger pyroptosis, a process regulated by mitochondrial oxidative stress. In the following sections, we will review recent data showing how dysfunctional mitochondria can control different types of immunogenic cell death relevant to neurodegeneration, such as necroptosis and metal ion-induced cell death.

### 4.1. Necroptosis

**Figure 5:**
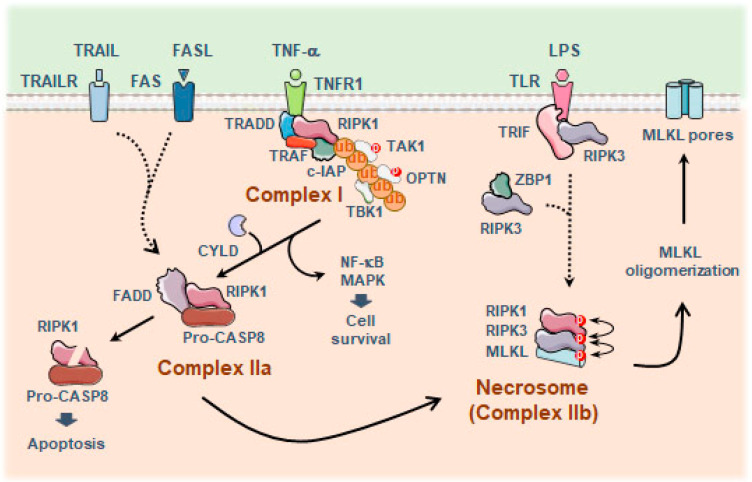
TNFR1-mediated survival and cell death pathways. Cell death signaling engaged by TNFα initiates with the adaptor TRADD protein that binds TNFR1 by its death domain and, in turn, serves as a signal to recruit RIPK1. Then, RIPK1 is polyubiquitinated by the combined action of TRAFs with c-IAPs and triggers the translocation of different ubiquitin-associated proteins and kinases, including OPTN, TAK1, and the serine/threonine-protein kinase TBK1. This signaling cascade promotes the stabilization of RIPK1 in complex I and the activation of pro-survival NF-κB and MAPK pathways. Conversely, inhibition of c-IAPs permits the removal of polyubiquitin chains of RIPK1 by the ubiquitin carboxyl-terminal hydrolase CYLD. RIPK1 is released from complex I and binds FADD and pro-CASP8 in complex IIa. TRAIL/TNFSF10 and FASL-mediated signaling can also participate in the assembly of complex IIa. Then, activated CASP8 can cleave the kinase domain of RIPK1, thereby triggering apoptosis; however, when CASP8 is deficient, RIPK1 remains active, and the signaling pathway is switched from apoptosis to necroptosis. Under necroptotic permissive conditions, RIPK1 interacts with RIPK3 through their RHIMs motifs, leading to the formation of a signaling platform, named necrosome (complex IIb). Upon necrosome assembly, RIPK3 forms homodimers, which leads to activating cis-autophosphorylation of T231/S232 in mouse and S227 in human RIPK3. Then, the kinase domain of RIPK3 binds to the C-terminal domain of MLKL and phosphorylates S345 in mice and T357/S358 in human MLKL, allowing the assembly of MLKL into oligomers. Ultimately, these MLKL polymers form pores in the plasma membrane that drive lytic cell death [156]. Abbreviations: CASP8, caspase 8; c-IAPs, cellular inhibitors of apoptosis; CYLD, CYLD lysine 63 deubiquitinase; FADD, Fas-associated death domain; FASL, Fas ligand; MAPK, mitogen-activated protein kinases; MLKL, mixed lineage kinase domain-like; NF-κB, nuclear factor kappa B; OPTN, optineurin; RIPK, receptor-interacting protein kinase; TAK1, transforming growth factor-β-activated kinase 1; TBK1, TANK-binding kinase; TRADD, TNFR1-associated death domain protein; TRAF, TNFR-associated factor; TRAIL/TNFSF10, TNF superfamily member 10; RHIM, RIP homotypic interaction motifs.

Necroptosis is a form of programmed necrosis mediated by the combined action of the receptor-interacting protein kinase 3 (RIPK3) and the pseudo-kinase mixed lineage kinase domain-like (MLKL) protein, with the participation of RIPK1 when death receptors are engaged [155]. The necroptotic molecular pathway has been most thoroughly studied in the context of the tumor necrosis factor receptor 1 (TNFR1) complex [156], although other members of the death receptor family, such as CD95/Fas and TRAILR, have also been implicated [157] (Figure 5).

Activation of RIPK3 has also been described independently of RIPK1, by binding to TIR-domain-containing adapter-inducing interferon-β (TRIF), a downstream mediator of TLRs [158], or the cytoplasmic viral DNA sensor Z-DNA binding protein 1 (ZBP1), which allows a rapid necrotic response upon viral infection [159] (Figure 5). Remarkably, activated RIPK3 and MLKL in immune cells have been shown to induce an inflammatory response independently of cell death by triggering the assembly of the NLRP3 inflammasome [160,161,162,163]. Subsequently, CASP8 has been indicated to directly cleave GSDMD and cause pyroptosis [164], thus further reinforcing the concept that different modes of inflammatory cell death display a considerable degree of interconnectivity.

Like other forms of programmed cell death, necroptosis is tightly regulated, with transcriptional changes, post-translational modifications, and elimination of necrosome members [165]. The autophagy machinery has been proposed as a molecular platform for necrosome assembly [166]. Additionally, autophagy controls the availability of RIPK1 and RIPK3 [167,168], and robust RIPK- and ROS-dependent necroptosis is observed after autophagy flux is chemically inhibited or when the lysosomal capacity is compromised [167,169,170]. Likewise, the ubiquitin E3 ligase Parkin, whose loss-of-function mutations are linked to autosomal PD, was recently identified as a negative regulator of necroptosis by leading to K33-linked polyubiquitination of RIPK3 and its proteasomal degradation [171]. Additionally, cellular extrusion of phospho-MLKL within extracellular vesicles has been proposed as a mechanism to downregulate MLKL activity and prevent necroptosis [172].

The occurrence of necroptosis has also been linked to mitochondrial function. Despite initial studies showing that mitochondrial depletion by mitophagy does not affect the kinetics of necroptosis [173], further works indicate that necrosome formation requires the production of mtROS [174,175]. Zhang et al. have identified three cysteine residues (C257, C268, and C586) in RIPK1 that can sense mtROS during necrosome execution [176]. These oxidative modifications activate RIPK1 autophosphorylation on S161, enabling RIPK1 to recruit RIPK3 and form a functional complex. Remarkably, at least in TNF-induced necroptosis, mitochondrial oxidative stress is exacerbated by RIPK3-dependent induction of the pyruvate dehydrogenase complex, resulting in enhanced aerobic respiration and high mtROS as a byproduct [177]. MLKL activity is also subjected to redox control by the thiol oxidoreductase thioredoxin-1, which maintains the monomeric MLKL in a reduced inactive state, thereby preventing disulfide bond-dependent polymer formation [178]. On the other hand, recent reports have confirmed the key role of mtROS in triggering necroptosis in experimental acute pancreatitis by a mechanism that requires p53-induced downregulation of the mitochondrial antioxidant enzymes sulfiredoxin and peroxiredoxin III [179]. In contrast, the mitochondrial membrane protein phosphoglycerate mutase family member 5 (PGAM5), whose deficiency impairs PINK1-mediated mitophagy and leads to a PD-like phenotype [180], has been reported to downregulate necroptosis by promoting mitophagy and reducing mtROS [181]. Interestingly, inflammasome activation, normally associated with GASDMD-dependent pyroptotic death, has been shown to shift toward necroptotic cell death in leucine-rich repeat kinase 2 (Lrrk2) G2019S mutant macrophages [182]. The presence of the disease-associated gain-of-function allele *Lrrk2^G2019S^* (a common genetic cause of PD) impairs mitochondrial function, resulting in high mtROS, which directs GSDMD to the mitochondria and activates the necroptotic pathway [182].

#### Necroptosis and Aging-Related Neurodegenerative Diseases

Emerging evidence underscores the significance of necroptosis in neurodegenerative processes [183]. In cell cultures and genetically engineered mouse models that recapitulate AD and PD, treatment with necrostatin-1, an inhibitor of RIPK1, has shown promise in reducing neuronal loss and improving cognitive functions [184,185,186,187]. Similar results have been obtained in subsequent studies inhibiting MLKL activity [188,189]. This aligns with observations of active necrosomes in the substantia nigra pars compacta of PD patients and in postmortem AD brains [187,190,191]. In AD, necrosome components tend to accumulate in granulovacuolar degeneration bodies [192], which are linked to tau-associated lysosomal disturbances [193]. Salvadores et al. described that neuronal necroptosis induction is triggered by Aβ-stimulated microglia [194]. Recently, using a human–mouse chimeric AD model, Balusu et al. found that human neuronal progenitor cells, when differentiated and transplanted into an Aβ-producing mouse, develop tau pathology and undergo necroptosis [195]. Their findings suggest a human-specific response to Aβ plaques, linked to the upregulation of the long non-coding RNA maternally expressed gene 3 (MEG3), which ultimately activates the necroptotic pathway. Hyperphosphorylated tau and Aβ oligomers have both been described to facilitate the formation of the necrosome complex [196,197,198], with the participation of mitochondria and the production of mtROS [199]. Moreover, activation of the necroptotic pathway has been consistently shown in preclinical models of PD, using toxins such as 1-methyl 4-phenylpyridinium (MPP+) and 6-hydroxydopamine (6-OHDA) that interfere with the activity of mitochondrial complex I of the respiratory chain [184,185,190], which further supports a key role of mitochondria in this process and underscores the potential of targeting mitochondrial pathways as a therapeutic strategy to mitigate necroptosis and neuroinflammation in neurodegeneration.

### 4.2. Metal Ion-Induced Cell Death: Ferroptosis and Cuproptosis

Transition metal ions, including iron (Fe), copper (Cu), zinc (Zn), and manganese (Mn), are involved in many biochemical processes essential for cell survival, and therefore, their levels are tightly regulated. However, under some conditions, excess redox-active essential metals such as Fe and Cu can induce hydroxy radical (·OH) via Fenton or Fenton-like reactions, causing cell damage and inflammation.

#### 4.2.1. Ferroptosis

**Figure 6:**
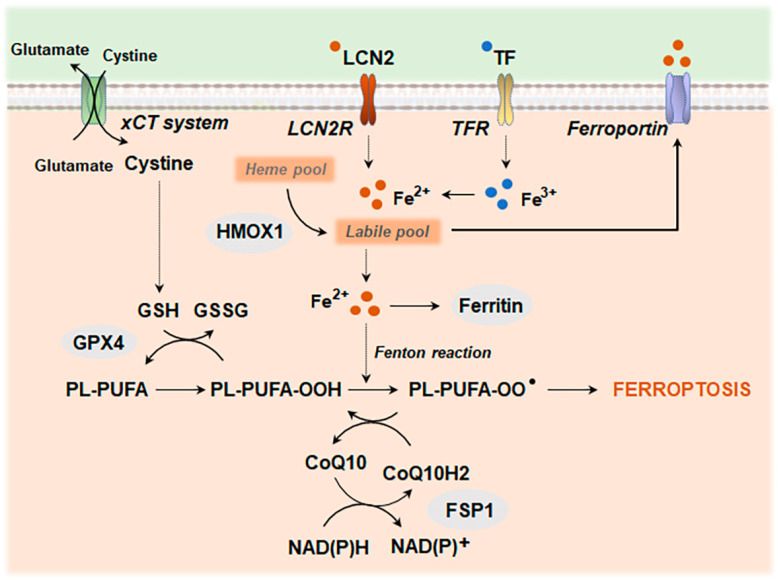
Core mechanisms of ferroptosis. TF and LCN2 transport extracellular iron into the cell through their respective receptors. Meanwhile, ferroportin is responsible for exporting intracellular iron to maintain iron balance. Inside the cells, HMOX1 breaks down heme to release free Fe^2+^, while ferritin, the iron storage protein, regulates intracellular iron levels to prevent iron overload. Ferrous iron (Fe^2+^) converts peroxides into free radicals through the Fenton reaction. This process leads to excessive lipid peroxidation and ultimately triggers ferroptosis. Ferroptosis can be prevented by two main antioxidant systems. One involves GPx4, which reduces lipid peroxides in a GSH-dependent reaction. The other is mediated by FSP1, which regenerates ubiquinone (CoQ10) to act as a trap for lipid peroxyl radicals. The cystine–glutamate antiporter SLC7A11 (xCT system) mediates the uptake of cystine, which is used for GSH synthesis. Abbreviations: CoQ10, coenzyme Q10; FSP1, ferroptosis suppressor protein 1; GPX4, glutathione peroxidase 4; HMOX1, heme oxygenase 1; LCN2, lipocalin 2; (PL-PUFA) phospholipids PUFA; SLC7A11, solute carrier family 7 member 1; TF, transferrin.

Dysregulation of iron metabolism and redox systems leads to the accumulation of intracellular lipid hydroperoxides, ultimately causing ferroptosis (Figure 6). Lipid peroxidation may kill cells directly by affecting cellular membrane integrity or indirectly through the generation of aldehydes, such as 4-hydroxynonenal (4-HNE) and malondialdehyde (MDA), which form adducts with different biomolecules and can act as a signal to drive cell death [200,201]. Mitochondria play a crucial role in the regulatory network of ferroptosis. Dysfunctional mitochondria-related alterations, including mitochondrial membrane depolarization, mtROS overproduction, and mtDNA release, can favor this type of cell death [202,203]. In particular, recent studies point to mtROS as an essential step in the activation of the ferroptotic pathway when the cystine–glutamate antiporter solute carrier family 7 member 1 (SLC7A11, also known as the xCT system) that supplies cystine for glutathione (GSH) synthesis or the glutathione peroxidase 4 (GPX4) are inhibited [202]. Concordantly, the inhibition of NRF2, which controls the expression levels of an array of antioxidant response element (ARE)-dependent genes, enhances the pathway, while mtROS scavengers prevent ferroptosis [202,203,204].

Ferroptosis has been described in many inflammation-related diseases, closely connected with the activation of NF-κB-driven inflammatory signaling pathways [205,206]. In a retinal degeneration mouse model, increased lipid peroxidation and ferroptosis have also been associated with a cGAS–STING-mediated inflammasome activation due to the autophagy and lysosomal dysfunction induced by iron homeostasis deregulation [207]. Likewise, NLRP3 inflammasome induction has been reported in a rat model of pulmonary hypertension after high mobility group box 1 (HMGB1) was released from ferroptotic cells, a critical DAMP that binds to TLR4 [208]. Both the lipophilic radical scavenger ferrostatin-1 and specific GPX4 activators can downregulate NF-κB signaling and suppress inflammatory conditions [209,210,211]. Furthermore, GPX4 and its ability to reduce lipid peroxides have been shown to downregulate GSDMD-mediated pyroptosis [212,213].

Iron overload is a pathological feature shared by multiple neurodegenerative diseases, including AD and PD [214,215,216]. Although the precise mechanism of iron-driven toxicity is unclear, there is evidence for the activation of the ferroptotic pathway. Increased endogenous levels of α-syn have been reported to sensitize dopamine neurons to ferroptosis [217]. A similar susceptibility to ferroptosis is displayed by cells carrying AD causal mutations of presenilins (PSEN) [218]. Astrocytes from AD patients and APPswe/PSEN1dE1 mice display elevated levels of aldehydes associated with high levels of NADPH oxidase 4 (NOX4) [219]. Upregulation of NOX4 in human astrocyte cultures promotes ferroptosis by affecting mitochondrial metabolism [219]. The role of mitochondria is further supported by different studies showing that strategies aimed at preserving mitochondrial function are effective against neuronal ferroptosis [220,221]. Interestingly, a recent work by Ryan et al. shows that iron overload causes an early shift in the microglial transcriptional state that overlaps with the transcriptomics displayed by microglia from postmortem PD brains [222]. The study further identifies SEC24 homolog B, COPII coat complex component (SEC24B), a protein involved in cargo packaging and intracellular trafficking, as the regulator of microglial ferroptosis and points to microglia as the main cause of ferroptosis-driven neurodegeneration [222]. The induction of ferroptosis as a triggering factor for neurodegeneration is gaining increasing interest and is beginning to be recognized as a bona fide target for future development of treatment and prevention strategies [223].

#### 4.2.2. Cuproptosis

**Figure 7:**
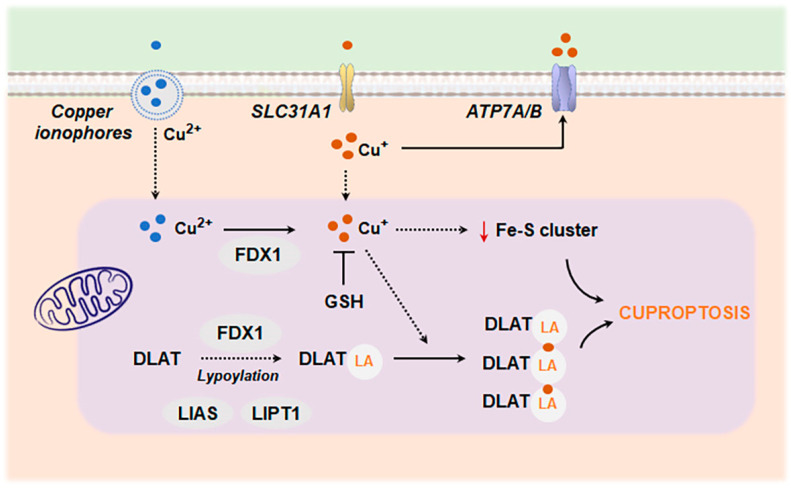
Molecular mechanisms of Cuproptosis. Extracellular copper can be transported within the cell via a copper ionophore (elesclomol, disulfiram, etc.). FDX1 reduces Cu^2+^ to Cu^+^, facilitating the release of copper ions in the mitochondrial matrix. FDX1 also binds to LIAS to promote lipoyl moiety generation. Then, LIPT1 transfers the LA to target proteins, such as DLAT. Lipoylated mitochondrial proteins (specifically DLAT) exhibit a high affinity for Cu^+^ binding, which causes their aggregation and loss of function. Moreover, excess of Cu^+^ reduces Fe-S cluster biosynthesis, destabilizes Fe-S cluster-containing proteins and contributes to overload Cu-mediated mitochondrial dysfunction and cell death. Copper importer SLC31A1 and exporters ATP7A/B can regulate the degree of cuproptosis sensitivity by controlling the intracellular concentration of copper ions. Also, GSH can serve as an endogenous copper chelator and protect against cuproptosis. Abbreviations: ATP7A/B, ATPase copper transporting alpha and beta; DLAT, dihydrolipoyl transacetylase; FDX1, ferredoxin 1; GSH, glutathione; LA, lipoic acid; LIAS, lipoic acid synthase; LIPT1, lipoyltransferase 1; SLC31A1, solute carrier family 31 member 1.

Copper is an important cofactor of several mitochondrial respiration complexes. Therefore, cells heavily dependent on mitochondrial respiration are especially vulnerable to mitochondrial copper accumulation. In physiological conditions, copper homeostasis is tightly regulated by chaperones (ATOX1), storage proteins (CuL), export mechanisms (ATP7A/B), and mitochondrial antioxidant defense or mitophagy [224]. Deregulation of these mechanisms can cause copper accumulation in mitochondria, which ultimately triggers a type of cell death known as cuproptosis. This type of cell death is regulated by ferredoxin 1 (FDX1), which reduces cupric (Cu^2+^) to cuprous (Cu^+^) ions. Cu^+^ binds to mitochondrial lipoylated proteins that participate in the tricarboxylic acid (TCA) cycle, specifically dihydrolipoyl transacetylase (DLAT), an enzyme involved in the formation of the pyruvate dehydrogenase (PDH) complex. The lipoyl moiety enables direct copper binding to these proteins, favoring their aggregation and loss of function. Also, Cu^+^ disrupts the synthesis of iron–sulfur (Fe-S) clusters, redox-active cofactors in the ETC [225] (Figure 7).

Cuproptosis has been related to cognitive impairment in mice exposed to copper overload [226]. Additionally, using integrated bioinformatics analyses and machine learning algorithms, recent studies have identified dysregulated cuproptosis-related genes (CRGs) linked to specific immune infiltration patterns in the substantia nigra of PD patients, suggesting that CRGs participate in PD progression through regulating immune cell infiltration and activity [227,228,229]. Similarly, two cuproptosis-related molecular clusters were defined in AD [230]. Functional analysis showed that cluster-specific and differentially expressed genes were closely related to various immune responses [230]. Aberrant intracellular copper accumulation may also modulate the microglial inflammatory response. Microglia cells exposed to Cu display an increased presence of inflammatory mediators such as NF-kB, fueled by elevated levels of mtROS [231,232,233]. Furthermore, it has been shown that the redox activity of Cu is necessary to properly activate the NLRP3 inflammasome [233,234]. In AD, mitochondrial oxidative stress elicited by chronic Cu overload exacerbates the impact of Aβ on microglial activation [235,236], promoting a shift toward degenerative gene expression signatures [237].

In recent years, several metal-chelating agents have been proposed as a potential therapeutic approach to restore metal ion balance in the brain and treat neurodegenerative diseases [238]. Prominent among them is a new generation of weaker chelating agents, known as metal protein-attenuating compounds (MPACs), which downregulate abnormal metal ion levels without causing damage to other essential metal-dependent processes. However, further research is still needed to improve their ability to cross the blood–brain barrier (BBB), understand their mode of action, and enhance their specificity, among other aspects.

## 5. Concluding Remarks and Future Perspectives

Collectively, there is compelling evidence that links an aberrant inflammatory response with the onset and progression of neurodegeneration. Immune system changes are particularly relevant in age-related neurodegenerative diseases where other alterations associated with aging, such as defective proteostasis and mitochondrial dysfunction, converge and may fuel neuroinflammation.

Preclinical studies have demonstrated the benefits of blocking NLRP3 inflammasome signaling. Inflammasome inhibitors reduce misfolded protein aggregates associated with neurodegenerative diseases and alleviate cognitive decline, suggesting that targeting the NLRP3 inflammasome could represent a novel therapeutic approach for neurodegenerative disorders. Various strategies to block this complex are under investigation, ranging from RNA-targeted therapies, nanobodies, and novel small molecule inhibitors [239]. However, a challenge for the field is the ability to translate findings from cellular and animal models of neurodegeneration into effective drug development for clinical use. Our recent studies have emphasized the importance of neuro–glia crosstalk in regulating inflammasome-mediated immune response [152], which should also be taken into consideration. Additionally, it is crucial to note that inflammasomes are necessary for a proper innate immune defense system. Therefore, finding a way to reduce NLRP3 activity without harming the immune system presents a significant challenge.

Beyond NLRP3 inhibitors, new therapeutic strategies focused on microglial modulation or cGAS–STING inhibitors are emerging to treat neurodegenerative diseases [240,241]. Most microglial interventions aim to shift their phenotype from proinflammatory or cytotoxic to more protective profiles, enhancing their phagocytic capacity, modulating their metabolism, or reducing the synthesis and secretion of exosomes and proinflammatory cytokines [240]. Recent results from preclinical and phase I clinical trials using AL002c, a TREM2 agonist antibody, have demonstrated good brain penetrance and tolerability, and its efficacy is currently being tested in early AD [242]. On the other hand, the type I IFN response can be ameliorated by using inhibitors of cGAS, STING, or TBK1 [243]. Although there are currently no clinical trials of cGAS–STING inhibitors in neurological diseases, a recent phase I trial with a cGAS antagonist (VENT-03) is ongoing, with the further aim of testing it in autoimmune diseases like lupus [244]. This new clinical approach, along with its extended use in preclinical studies in neurological disorders [241], positions modulators of the type I IFN pathway as potential therapeutic targets for controlling neuroinflammation.

One plausible therapeutic intervention could involve antioxidant supplementation, given the prominent role of oxidative stress in regulating neuroinflammation at different levels. In this sense, many efforts have been focused on exploring the properties of natural and synthetic antioxidant compounds. However, despite their potential, the clinical use of antioxidants is still limited due to issues such as low bioavailability, easy degradation, and poor solubility in water. To address these problems, nanomedicine has been proposed as an innovative approach to deliver antioxidants to the brain [245]. Nanoparticles can encapsulate the drug, protecting it from degradation and increasing its bioavailability and pharmacokinetic properties. In addition, nanoparticles can be functionalized with specific ligands to target particular organelles, such as mitochondria [246,247,248]. The rapid advancement of nanomedicine offers the possibility of designing tailored nanocarriers for targeted antioxidant delivery to the brain, paving the way for innovative therapeutic solutions.

## Data Availability

Not applicable.
